# Changes in urine headspace composition as an effect of strenuous walking

**DOI:** 10.1007/s11306-015-0813-8

**Published:** 2015-05-31

**Authors:** Devasena Samudrala, Brigitte Geurts, Phil A. Brown, Ewa Szymańska, Julien Mandon, Jeroen Jansen, Lutgarde Buydens, Frans J. M. Harren, Simona M. Cristescu

**Affiliations:** 1 Institute Molecules & Materials, Life Science Trace Gas Facility, Radboud University Nijmegen, 6500 GL Nijmegen, Netherlands; 2 Institute Molecules & Materials, Department Analytical Chemistry, Radboud University Nijmegen, 6500 GL Nijmegen, Netherlands; 3 TI-COAST, Science Park 904, 1098 XH Amsterdam, Netherlands

**Keywords:** Urine, Walking, Acetic acid, PTR-MS, Acetone

## Abstract

The present investigation uses proton transfer reaction mass spectrometry (PTR-MS) combined with multivariate and univariate statistical analyses to study potential biomarkers for altered metabolism in urine due to strenuous walking. Urine samples, in concurrence with breath and blood samples, were taken from 51 participants (23 controls, 11 type-1 diabetes, 17 type-2 diabetes) during the Dutch endurance walking event, the *International Four Days Marches*. Multivariate analysis allowed for discrimination of before and after exercise for all three groups (control, type-1 and type-2 diabetes) and on three out of 4 days. The analysis highlighted 12 molecular ions contributing to this discrimination. Of these, acetic acid in urine is identified as a significant marker for exercise effects induced by walking; an increase is observed as an effect of walking. Analysis of acetone concentration with univariate tools resulted in different information when compared to breath as a function of exercise, revealing an interesting effect of time over the 4 days. In breath, acetone provides an immediate snapshot of metabolism, whereas urinary acetone will result from longer term diffusion processes, providing a time averaged view of metabolism. The potential to use PTR-MS measurements of urine to monitor exercise effects is exhibited, and may be utilized to monitor subjects in mass participation exercise events.

**Electronic supplementary material:**

The online version of this article (doi:10.1007/s11306-015-0813-8) contains supplementary material, which is available to authorized users.

## Introduction

Due to a persistent absence of phenotypic symptoms during progression, many chronic diseases like diabetes can remain undiagnosed during the early stages of development. Even after diagnosis, the suitability and effectiveness of medication or lifestyle interventions (diet, exercise etc.) are difficult to interpret, yet critically important for managing the treatment of a chronic disease. Personal omics profiling (POP) aims to tackle these challenges by scanning individuals in detail to identify the genetic basis for disease risk or treatment efficacy, and using this knowledge to enable daily monitoring with post-genomic technology (Chen et al. 2012; Roukos 2008). Large-scale implementation of this approach would however put a disproportionate burden on healthcare resources, as this requires extensive use of highly advanced technology. Likewise, this would require considerable effort of the healthy examinee, as he/she would need to regularly visit the appropriate medical infrastructure for a relatively invasive procedure; typically the provision of blood samples. Although highly sensitive to detect and monitor a broad range of potential diseases, in its current state POP is too impractical to be viably implemented.

A truly feasible implementation would be to collect samples in locations where many members of the target group are voluntarily present. Several samples (breath, urine) can then be obtained considerably less-invasively, without requirement for specifically trained personnel. However, metabolomics technology is put to the challenge for such scenarios, as the sheer number of samples generated on such highly populated locations requires rapid analysis and the diluted state of specifically volatile samples requires highly sensitive analysis platforms.

The volatile organic compounds (VOCs) emitted from the skin (Turner et al. 2008), exhaled in breath (Buszewski et al. 2007; Schwarz et al. 2009; Lourenço and Turner 2014) or present in urine (Huang et al. 2013; Mochalski et al. 2012) have been well characterized in healthy people in the literature (Costello et al. 2014). Changes in the concentration of specific VOCs can indicate particular diseases (Smolinska et al. 2014) and changes in metabolic state, such as nitric oxide in airway inflammation (Barnes et al. 2010) or acetone for diabetes (Storer et al. 2011), and as such they are considered biomarkers (Mazzatenta et al. 2013). Proton transfer reaction mass spectrometry (PTR-MS) (Lindinger et al. 1998; Blake et al. 2009) is a popular, highly sensitive, on-line tool used to measure concentrations of excreted metabolites, particularly in breath (Herbig et al. 2009; Schwarz et al. 2009) but also urine (Pinggera et al. 2005). The technique allows rapid analysis of VOCs, due to its online capability. Accurate measurements of VOC concentrations are possible in seconds. The present study aims to use PTR-MS coupled with multivariate and univariate statistical techniques to investigate potential biomarkers for exercise altered metabolism in urine. In a previous publication, breath acetone, which was measured with proton transfer reaction ion trap mass spectrometry, correlated positively with both non-esterified fatty acids and beta-hydroxybutyrate (BOHB) (markers in blood for fatty acid metabolism), providing real-time information on fat burning (Samudrala et al. 2014). We now examine the relation between urine acetone and breath acetone and evaluate the VOCs in urine as possible biomarkers for the effect of 4 days strenuous walking, as well as their interplay with medicated type 1 and type 2 diabetes mellitus.

## Materials and methods

### Subjects

All subjects participated in the International Four Days Marches, July 2012, an annual walking event in Nijmegen, the Netherlands, organized by the Dutch Walking Organization (KNBLO-NL). In total 51 participants gave urine samples. Among them 23 were control (CT), 11 type-1 diabetes mellitus (T1DM) and 17 type-2 diabetes mellitus (T2DM). Participants included 28 males and 23 females with an age range of 25–85 years. Depending on their age and gender, participants walked 30, 40 or 50 km per day. The details of the subjects who participated in this study are shown elsewhere (Samudrala et al. 2014). This study was approved by the Medical Ethical Committee of the Radboud University Nijmegen Medical Centre. Informed consent was obtained from all individual participants included in the study, and the study was conducted in accordance with the Declaration of Helsinki.

### Urine samples

Urine samples were collected twice a day for 4 consecutive days from each subject, once in the morning, prior to walking (between 3:30 am and 8:30 am) and another at the end of the walk (between 10:30 am and 17:30 pm).

After collection, the samples were transported via a cooler (~6 °C) to a storage freezer of temperature −80 °C. Samples were without any centrifuge separation and without any anti-bacterial additives. The samples were later defrosted, separated into two vials and refrozen. One vial was used for VOCs analysis with PTR-MS and the other was used for creatinine measurements. Creatinine was measured via an enzymatic method using an ARCHITECT clinical chemistry analyzer (Abbott Laboratories, Abbott Park, Illinois, USA).

### VOCs analysis from urine headspace

Urine measurements were performed with an in-house built proton transfer reaction mass spectrometer (PTR-MS). A detailed description of this instrument has been given elsewhere (Steeghs et al. 2004) and the instrument bears a strong similarity to commercially available devices (Lindinger et al. 1998). Here, we give only a brief introduction to the technique. Proton transfer reaction is a soft ionization method in which VOCs with molecular mass (M) are ionized by the transfer of a proton from a hydronium ion (H_3_O^+^), and are detected at mass M + 1 using a quadrupole mass spectrometer. Signal intensity of the hydronium ion and the protonated water cluster (*m/z* 19 and *m/z* 37) are typically measured for normalization. Apart from them, 38 other ions were measured, including acetone (*m/z* 59); the dwell time for each ion was 0.2 s.

Samples to be measured by PTR-MS were removed from the freezer the night before the measurements to defrost at room temperature (~20 °C). The following day the instrument was calibrated and samples prepared and measured. 10 ml of urine was transferred from the vial to a glass cuvette with a 40 ml available volume. The closing lid of the glass cuvette was sealed in place with a metal fastening clip and an O-ring, with one gas inlet and one gas outlet port to allow sampling. A constant flow of 2 l/h of air was passing through the cuvette to flush the head space. The outlet for this flow was side sampled by the PTR-MS at 1.5 l/h. For the duration of the measurement the samples remained at room temperature. The intensity of the VOC signal was observed to decrease with time, due to the continuous flushing of the headspace. Therefore, to prevent the concentration declining, the headspace was flushed and measured for 4 min only for each sample. Beyond this time the headspace concentration drop becomes unacceptable (more than 18 % drop). Three samples were measured sequentially using an automatic valve system (van Dam et al. 2012) to switch between each cuvette. An example of this measurement is shown in supplementary material: S1, with a repetitious measurement for method validation. The product ion intensities, measured in counts per seconds (cps) were normalized to reagent ion signal, which gave intensities in normalized counts per second (ncps). These were further normalized to creatinine concentrations (mmol) to give final units of ncps mmol^−1^. This allowed the detected signals to be normalized with respect to the level of biological dilution present in the sample (Amann and Smith 2013). For the duration of analysis, the transfer lines from the valves to the PTR-MS were heated up to 55 °C to prevent condensation.

The consistency of the analysis was checked by calibrating each day, before the start of the experiment with a standard gas mixture consisting of methanol (*m/z* 33), acetaldehyde (*m/z* 45), acetone (*m/z* 59), isoprene (*m/z* 69), benzene (*m/z* 79), toluene (*m/z* 93), *O*-xylene (*m/z* 107) and alpha-pinene (*m/z* 137), each in 1000 ppbv ± 5 % (ppbv = part per billion volume) in a nitrogen dilution gas (Linde, Dieren, the Netherlands). The calibration was performed using different concentrations, from 35 to 1000 ppbv obtained by dilution of the standard mixture with nitrogen gas.

### Statistical analysis

The data was first analyzed using a multivariate technique allowing the significance of all ions to be assessed. Univariate analysis was then repeated for those ions of highest significance to the multivariate model. Finally, the *m/z* 59 product ion of acetone was analyzed univariately for comparison with breath. The methods of univariate and multivariate analysis used in this analysis are explained below.

### Multivariate analysis

Analysis with multilevel partial least squares discriminate analysis (M-PLS-DA) (van Velzen et al. 2008; Szymańska et al. 2012) was carried out to study the effects of exercise from a multivariate perspective. Variation in the data contains an inter-individual part and an intra-individual part. The inter-individual subject variation describes the difference between the subjects, which is unrelated to the effect of exercise. In this study each subject participated both before and after exercise (and therefore serves as his/her own control), allowing for an intra-subject evaluation of the effect of exercise. In order to study the effect of exercise on the different groups, a separate M-PLS-DA model was built for each of the three groups (T1DM, T2DM and CT). The data were auto-scaled before analysis by M-PLS-DA. To evaluate the exercise effect on the different days, separate M-PLS-DA models were built for each of the days, taking all the groups together. The models aim to discriminate between before and after walking, and allow for identification of those ions that are important for this classification. Thirty-three ion signals in the range of *m/z* 33–*m/z* 117 were chosen for this analysis from the forty that were measured. Five ions were discarded because their count rate fell consistently below the detection limit of the instrument.

As a measure of model performance, the area under the receiver operator curve (AUROC) was calculated. The AUROC represents a ratio between the number of true positives and the number of false positives in the classification, where an AUROC of 1 represents perfect classification (Zweig and Campbell 1993). All M-PLS-DA models included double cross validation (van Velzen et al. 2008; Smit et al. 2007; Westerhuis et al. 2008). The optimal number of latent variables was chosen with a fourfold single cross validation. A subsequent fivefold double cross validation was used to assess the model performance on a test set. The statistical significance of the model performance was assessed by a subsequent permutation analysis with 1000 realizations. To evaluate which ions played an important role in this classification, the rank products of the variables in the model are used. All variables were ranked according to their PLS regression coefficients. The variables with the lowest rank products are the ones with the largest discriminative capability. The significance of the ranks was assessed by comparing the variable ranks of the model to those in the 1000 permutation tests, and a *p* value was assigned accordingly. Results are represented in a radar plot, where each variable is presented on one of the radial axes (Saary 2008). Against each axis, the value 1 − *p* is plotted, such that tests with lower *p*-values are positioned toward the outside of the radar field, and higher *p*-values are positioned toward the middle of the graph.

### Univariate analysis

The normality of the data distribution was evaluated using a Lillefors test. All the ion signals measured from the urine headspace were found to have a non-normal distribution, such that non-parametric tests were used for all subsequent analyses. A Wilcoxon signed rank test was used to determine the effect of exercise on each individual mass. To evaluate the effect of time—the change of concentration over the 4 days—the Friedman test was used because of its ability to handle multiple attempts. Both the Wilcoxon signed-rank test and the Friedman test are ‘paired’ tests, comparing data from the same subject over different settings. Associations between the intensity of two ion species were evaluated using Spearman correlations. Any *p*-values < 0.05 were considered significant and *p* < 0.1 were considered trend to be significant. All statistical analyses were performed in MATLAB (version 2014a, The Mathworks, Natick, Massachusetts, USA). Boxplots were made in Origin (version 9.0, Origin lab, Northampton, Massachusetts, USA).

## Results

First, the effect of exercise on the detected VOCs is shown and studied as a whole using multivariate analysis. After that, possible identification and univariate analysis of significant ions is evidenced. Finally, the effect of exercise on headspace acetone is evaluated by univariate analysis.

### Multivariate analysis of the full data-set

Multivariate analysis was used to study the effect of exercise on detected ions and consequently emitted compounds from the urine headspace. A different classification model to extract the effect of exercise was made for each of the three groups, and the model performances are shown in Table 1(a).

**Table 1:** The ability of the models used to distinguish between before and after exercise is shown separately for (a) each group, for all 4 days together, and for (b) each day, for all three groups together

(a) Model performance per group			(b) Model performance per day		
Group	AUROC	*p* value	Day	AUROC	*p* value
CT	0.87	<0.001	1	0.63	0.012
T1DM	0.70	0.004	2	0.64	0.066
T2DM	0.81	<0.001	3	0.90	<0.001
			4	0.94	<0.001

**Fig. 1:**
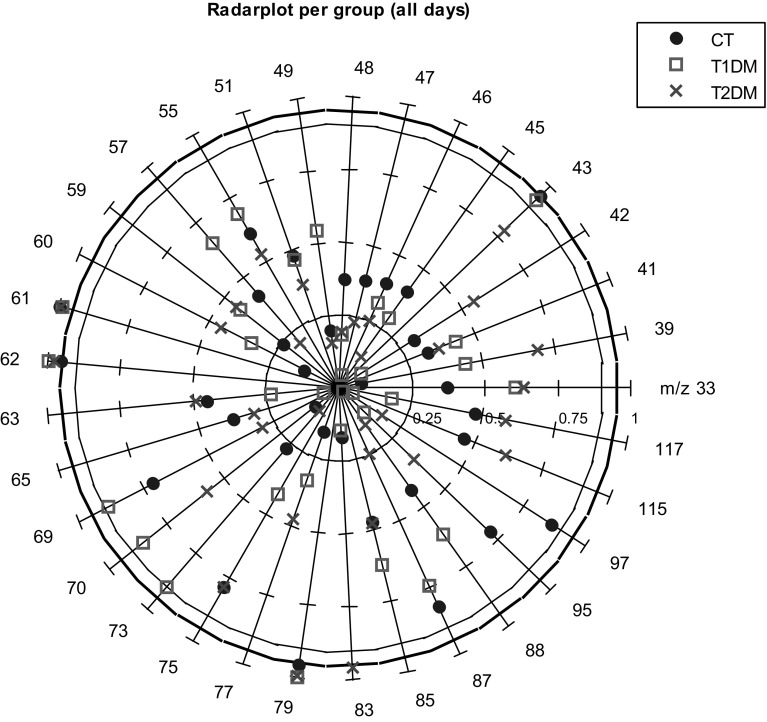
Radar plot per group showing the variable (*m/z*) contribution to the M-PLS-DA model classifying between before and after exercise

In all three groups, M-PLS-DA is able to extract a higher significant exercise effect than classification of a randomly permuted dataset. The highest model performance is achieved for the CT group, followed by T2DM, as can be seen by comparing the AUROC in Table 1(a). The contribution of each mass (*m/z* value) to the classification model is represented in a radar plot in Fig. 1. All three groups are represented in the same figure, allowing direct comparison of the variable’s importance to each of the models generated from a particular group. The outermost, bold black circle of the graph indicates the 5 % significance level. Values on and outside of this circle are considered significant. The circle drawn with a thinner line indicates the 10 % significance level. Ions near to the circumferences of these two circles are of particular importance.

Considering the 33 ions involved in this analysis, three ions: *m/z* 61, *m/z* 62 and *m/z* 79 showed significant contributions in discriminating the effect of exercise for all groups. In addition to these ions, *m/z* 43 is contributing for CT and T1DM groups, *m/z* 73 only for T1DM and *m/z* 83 for T2DM. This indicates that these ions are strongly involved in forming a multivariate model to discriminate the effect of exercise in the three groups of subjects.

To evaluate the effect of exercise over the course of the 4 days, M-PLS-DA was used to classify between before and after exercise on each of the days. Four different models were made, and their performances are shown in Table 1(b).

**Fig. 2:**
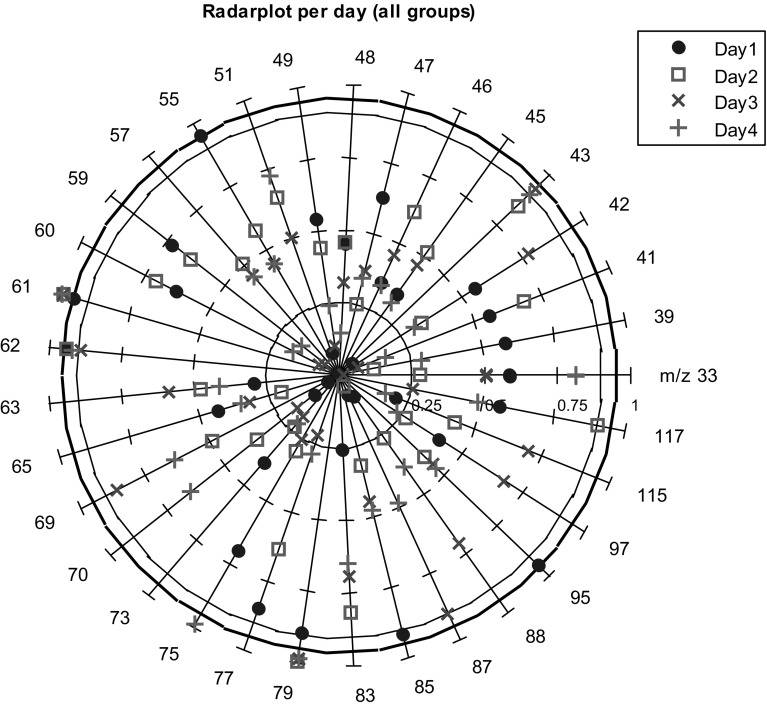
Radar plot per day showing the variable (*m/z*) contribution to the M-PLS-DA model classifying between before and after exercise

Discrimination of before and after exercise was significantly relevant for all days, except for day 2. The ability to discriminate was greatest for day 4, followed by day 3. This shows that the perceived metabolic effect of exercise increases from day to day, and is especially high on the last 2 days of the marches. The significance of the variable contribution is shown in a radar plot in Fig. 2.

The ions at *m/z* 61, 62 and 79 are contributing significantly to the discriminative model on all 4 days. Other ions exhibit significance on some but not all days and these are summarized in Table 2. The possible compounds/fragments for these ions measured in urine headspace are shown also in Table 2 with reference to the previously reported urine analysis and PTR-MS ion identification techniques.

**Table 2:** Ions from the headspace of urine measurements that showed significant contribution in discriminating the effect of the exercise during the 4 days walking program

*m/z*	Possible protonated -monomer (M) -fragment (F) -cluster (C) -isotope (I)	Effect of exercise on each group (Fig. 2) CT/T1DM/T2DM	Effect of exercise on each day (Fig. 3) Day 1/Day 2/Day 3/Day 4	Inter-comparison with previous related NMR and MS studies	Volatile compound identification^a^
43	Acetic acid; *CAS: 64*-*19*-*7* (F)	CT, T1DM	Day 3, Day 4	Enea et al. (2010)	NIST-MS*; FCA
55	Water cluster (C) Hexanoic acid; *CAS: 142*-*62*-*1* (F) Phenol; *CAS: 108*-*95*-*2* (F)		Day 1	Brown et al. (2010), Huang et al. (2013), Troccaz et al. (2013)	NIST-MS*
61, 62, 79	Acetic acid (M, I, C)	CT, T1DM, T2DM	Day 1, Day 2, Day 3, Day 4	Enea et al. (2010)	ICA; CCA
73	Succinic acid; *CAS: 110*-*15*-*6* (F) Hexanoic acid (F)	T1DM		Deja et al. (2013), Huang et al. (2013)	NIST-MS*; CID
75	Methional; *CAS: 3268*-*49*-*3* (F)		Day 4	Troccaz et al. (2013)	NIST-MS*
83	Hexanoic acid (F)	T2DM		Huang et al. (2013)	NIST-MS*; CID
85	3-Hydroxy butyric acid; *CAS: 300*-*85*-*6* (F) Succinic acid (F)		Day 1	Deja et al. (2013)	NIST-MS*
87	3-Hydroxy butyric acid (F) Hexanoic acid (F)		Day 3	Deja et al. (2013), Huang et al. (2013)	NIST-MS*; CID
95	Dimethyl disulfide; *CAS: 624*-*92*-*0* (M)		Day1	Troccaz et al. (2013)	NIST-MS*
117	Hexanoic acid (M)		Day2	Huang et al. (2013)	NIST-MS*; CID

Possible identification of these ions in correspondence with the related literature and by some additional identification methods

Star (*) represents that the ion is tentatively identified

^a^NIST-MS, Identification by comparison with NIST mass spectrum (NIST 2011); ICA, identification by isotopic correlation analysis; CCA, cluster correlation analysis, using clusters formed due to the reaction with water; FCA, fragment correlation analysis, correlation between potential fragment and monomer ions; CID, suggested fragments from experiments performed by Ion trap mass spectrometer with the pure compound

The signals at *m/*z 61, 62 and 79 were consistently significant in both multivariate analyses. The signal observed at *m/z* 61 showed a correlation with *m/z* 62 (data not shown), relating to its ^13^C (1.1 %) carbon isotope with a slope value of 2.6 %; which is close to the theoretically predicted 2.3 % for a two carbon ion. This strongly suggests that *m/z* 62 is due to the ^13^C isotope of *m/z* 61 and that *m/z* 61 contains two carbon atoms. The ion signal observed at *m/z* 79, was found to have a significant contribution for prolonged exercise. The signal of *m/z* 79 was higher after exercise than before on day 2, 3 and 4. The correlation between *m/z* 61 and *m/z* 79 is R^2^ = 0.88. Therefore, *m/z* 79 may be the single hydrate cluster of *m/z* 61 (61 + 18 = 79). These correlations, along with knowledge of PTR-MS product ion identification imply that *m/z* 61, *m/z* 62 and *m/z* 79 are from the same compound. We propose that they are all product ions of acetic acid, an identification that we base on putative annotation, (level 2) as described by Sumner et al. (2007). Using this identification, no additional information is provided by treating these ions separately. Therefore, univariate analysis of acetic acid is shown from here on by univariate analysis of the ion at *m/z* 61 and the effect of exercise on this ion is verified.

### Univariate analysis of acetic acid

**Fig. 3:**
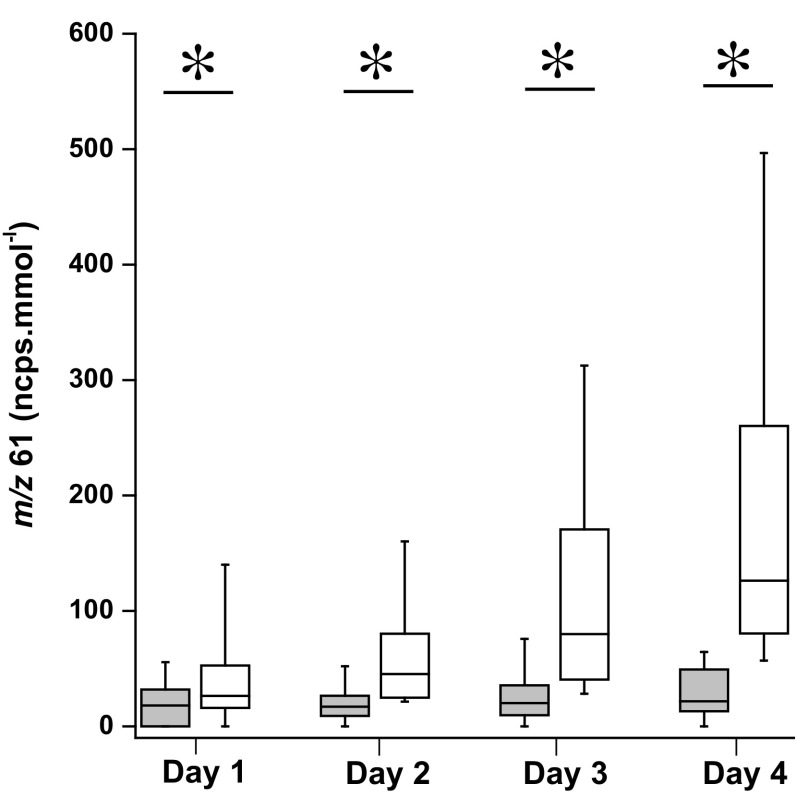
Urine headspace *m/z* 61 concentrations measured before and after exercise over 4 consecutive days for all participants. Data are displayed as box plots showing the median, interquartile ranges (25, 75 %); whiskers indicate the 10–90 % values. *indicates *p* < 0.05

The multivariate analysis showed that acetic acid significantly contributed to the models and allowed for the effect of prolonged exercise to be discriminated. Therefore, this ion was analyzed by univariate analysis. In Fig. 3, box plots are shown for acetic acid for all the 4 days, including all subjects. There is a significant effect due to exercise on acetic acid for all days; with *p*-values, 0.03 on day 1 and <0.001 for days 2–4. Both univariate and multivariate analysis highlighted the significance of this ion with respect to an effect of exercise, therefore it is confirmed that this ion is a possible marker for the effect of exercise in urine headspace.

Apart from these ions, other ions were putatively annotated (level 2) (Sumner et al. 2007) based on previous identification in similar studies shown in Table 2; measured urine VOCs as an effect of exercise (Enea et al. 2010) or diabetic markers (Deja et al. 2013) or markers for other disorders (Huang et al. 2013; Troccaz et al. 2013).

In addition to the acetic acid signal, significant ions are observed that may be product ions of hexanoic acid. However the signal of either product ion or fragment ions didn’t appear on each day in every group. This could be due to other ionic species being present at the same *m/z* values.

### Univariate analysis of acetone (*m/z* 59)

**Fig. 4:**
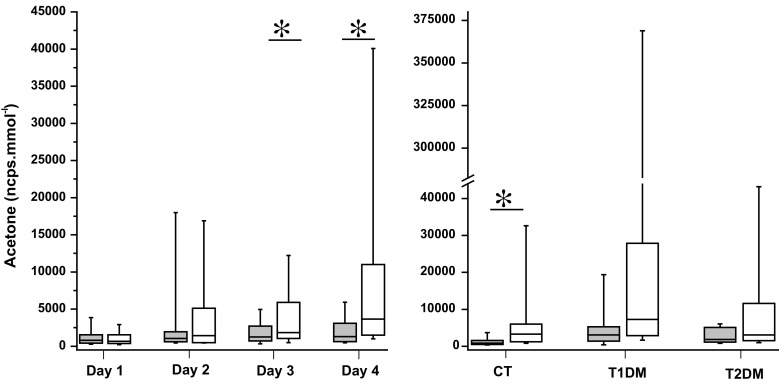
Univariate analysis of acetone: *Left panel* Urine headspace acetone concentrations measured before and after the walk over 4 consecutive days for all participants. *Right panel* Headspace acetone concentrations measured before and after the walk in CT, T1DM, and T2DM for 4 days. Data are displayed as box plots showing the median, interquartile ranges (25 %, 75 %); whiskers indicate the 10–90 % values. *indicates *p* < 0.05

Wang et al. (2008) reported finding an equilibration between urine acetone and breath acetone in two subjects, implying a correlative relationship. In the present study, breath concentrations and urinary headspace concentrations of acetone were checked for correlation, but no correlation was observed. A strong correlation for acetone concentration in urinary headspace with exercise was reported by Orhan et al. (2004). Acetone is obviously detected using PTR-MS, with a product ion observed at *m/z* 59. In the left panel of Fig. 4, the effect of exercise is shown on all participants with respect to the variation of acetone levels before and after exercise for the 4 consecutive days. For day 1 and day 2 there is no significant effect of exercise on measured headspace acetone (*p* = 0.46; *p* = 0.33). However, on the last 2 days, day 3 and day 4 the effect due to prolonged exercise is significant, with *p*-values 0.049 and <0.001 respectively. The effect of exercise on CT subjects is significant (*p* < 0.001), but not on T1DM and T2DM subjects (Fig. 4, right panel). In contrast to our previous experiments with breath acetone (Samudrala et al. 2014); there is a significant, exercise effect on acetone concentration in urine headspace when judged as a function of time.

## Discussion

Multivariate analysis revealed which measured ion signals contributed significantly to discriminating the effect of exercise. These results are validated using cross-validation and permutation testing, confirming the validity of the analysis for this data. The low number of samples is not optimal, and the results of the multivariate analysis should be confirmed in a larger cohort. These ion signals are shown in Table 2 with putative annotations. In PTR-MS, identification of ions is difficult and there is no library for the compound identification. However, tentative identification can be made depending on an ion’s fragmentation pattern and/or using isotopic ratios (Crespo et al. 2012) or comparing with the NIST mass spectrum (NIST 2011).

Acetate is a product of fatty acid metabolism. It can either convert into acetyl-CoA and participate in the Krebs cycle to produce energy, or with two molecules of acetate it can form acetoacetate which further converts into acetone due to decarboxylation (Miekisch et al. 2004). Xu et al. (2014) have recently raised the level of interest in acetate in relation to training by showing its importance in the mammalian stress-response to hypoxia. In addition, Fushimi et al. (Fushimi and Sato 2005; Fushimi et al. 2001) have shown that increased dietary acetic acid can stimulate glycogenesis, leading to increased glycogen recovery in skeletal muscles. Going on to conclude that increased dietary intake of acetic acid may be beneficial in glycogen recovery post-exercise.

In this study, evidence is presented that acetate in urine may be used to monitor the effect of subsequent days intense walking. Using breath analysis, of the same subjects, acetone was observed as a direct reflection of burning fat as an effect of walking (Samudrala et al. 2014). In univariate analysis, it is clearly evidenced that for each of the 4 days of exercise, acetate levels in urine are raised by the exercise, similar to the acetone behavior in breath analysis of the same cohort.

Two other ions, *m/*z 43 and *m/z* 79 could be the fragment and monohydrate cluster of acetic acid, drawing on evidence from the NIST chemistry web book and correlation analysis. As mentioned earlier, ketone bodies can be detected in urine samples. One of the ketone bodies that has been shown by Samudrala et al. (2014) to have a correlation with breath acetone is BOHB. In the present study, *m/z* 83 and *m/z* 87 showed contribution to the model on day 1 and day 3, respectively. These could be ion fragments of protonated BOHB in comparison with NIST mass spectrum. Previous studies also showed the occurrence of this compound in the urine of diabetic subjects (Deja et al. 2013) and due to physical exercise (Enea et al. 2010). Since these two ions are tentatively identified in this study, there is a possibility that several other compounds can have the same ion as a fragment. However, the ratio of these fragment ions will vary depending on the parent ion. Protonated hexanoic acid (*m/z* 117) has a major fragment ion at *m/z* 87. There are also minor fragment ion peaks at *m/z* 73, *m/z* 83 and *m/z* 55. Though these ions were highlighted on different days in different groups of people there could be a possibility that the origin of these ions is from the same parent molecule, hexanoic acid. Interference from other ions at the same mass may be confusing the analysis of hexanoic acid, for example when considering an ion at *m/z* 55 in PTR-MS studies, the H_3_O^+^·2H_2_O water cluster is a habitual concern (Brown et al. 2010).

Hexanoic acid is a medium chain fatty acid linked to fatty acid metabolism. Hexanoate has been observed in elevated levels in blood plasma of humans with T1DM, and rats with T1DM and T2DM under oxidative stress as a result of lipid peroxidation (Januszewski et al. 2005). This elevation in blood plasma could explain why the product ions at *m/z* 73 and 83, tentatively attributed to hexanoic acid, contribute significantly to the M-PLS-DA model shown in Fig. 1 only for T1DM and T2DM, respectively.

Huang et al. (2013) found that hexanoic acid is an important biomarker for the discrimination between patients with gastro esophageal cancer measured from urine headspace, along with another marker, acetic acid. Acetate and hexanoate are both transported through the cellular membrane by monocarboxylate transporter (MCT) proteins. These proteins have been shown to be altered by endurance training, and were studied with relation to possible up-regulation of the MCT-1 protein leading to improved clearance of lactate (Opitz et al. 2014). An increase in clearance rates for hexanoate and acetate would make a plausible explanation for the increase in acetic and hexanoic acids seen in urine. That a link exists between acetate, hexanoate and lactate is also of note because lactic acid is a commonly used marker for muscular activity (Gladden 2004). A correlation was looked for between hexanoic acid and acetic acid in this study, however no such correlation was found.

Urine is an odorous biological liquid. Therefore, the presence of odor compounds like sulfides are possible, due to the presence of different bacteria. A recent study by Troccaz et al. (2013) showed that dimethyl sulfide, trimethylamine, dimethyl disulfide, methional, and some phenols are responsible for this odor due to several bacterial interactions. However, the presence of these bacteria may vary from person to person, due to the changes in body conditions and the amount of by-products the body is producing for excretion. This is the reason that methional and dimethyl disulphide are mentioned in Table 2 as possible compounds.

Urine analysis is a non-invasive method for investigating integrated responses after application of stress/exercise on humans (Enea et al. 2010). However, only a few studies have reported the impact of physical exercise on urinary headspace VOCs (Orhan et al. 2004; Pechlivanis et al. 2010; Enea et al. 2010), while others report the effects of physical characters such as gender, age, and diurnal variation (Slupsky et al. 2007). A recent study on urinary headspace by Enea et al. showed that metabolomics is a promising tool to gain insights into changes induced by short term, intense physical exercise, with measurements made using proton NMR spectroscopy (Enea et al. 2010). In their study they observed the changes in the creatinine, lactate, pyruvate, acetate, BOHB and hypoxanthine due to the effect of short term intensive exercise.

This study represents the first measurements of the headspace of urine samples using PTR-MS to investigate the effect of prolonged exercise. Pinggera et al. (2005) have used PTR-MS for urinary measurements of acetonitrile in smokers, although they did not look for correlation with breath acetonitrile; they did find correlation between acetonitrile and smoking behavior. Using SIFT-MS, Wang et al. (2008) claimed that acetone was equilibrated amongst the body fluids by comparing acetone in breath with urinary headspace measurements of healthy volunteers. This claim is made on only a very limited cohort size of two volunteers. The data which we present did not show a correlation between urinary acetone and breath acetone.

Metabolites produced in the body as a result of different metabolisms are diffused from blood into urine and breath for further elimination. Breathing rate is typically 15–20 times per minute for adults and urination frequency is typically 6–7 times per 24 h (Nitti 2002). This variation in timing means that breath analysis provides insight into metabolic changes at the moment the sample is taken, whereas metabolites in urine samples result from a longer term diffusion process. This is the expected reason for the difference in the behavior of urinary acetone and breath acetone as an effect of prolonged exercise.

Samples of urine collected by this study were not treated with any antimicrobial agent, such as sodium azide. Samples were also not immediately deep-frozen. For these reasons, the notion that bacterial growth or metabolic degradation may have occurred in the sample and could have contributed compounds to the VOC profile cannot be discounted. In future studies it is recommended to add an antibacterial agent (Orhan et al. 2004; Slupsky et al. 2007) and to deep-freeze samples immediately after acquisition, as suggested in a review article by Want et al. (2010). Typical inter-day and intra-day variations of urine headspace samples measured with PTR-MS were not evaluated for this study. Diurnal variation has been analyzed by Slupsky et al. (2007) using NMR spectroscopy. A careful understanding of biological variation under control conditions is useful for assessing the importance of observed variation in non-control groups.

Previous studies reported mass spectrometry based techniques in measuring urine headspace measurements for different applications (Smith et al. 1999; Wahl et al. 1999; Huang et al. 2013). However, most of the studies use NMR spectroscopy to analyze urine samples. Metabolomics is a rapidly expanding field in recent years, due in part to the advancement of NMR. This technique detects a wide range of compounds with easy, straightforward quantification. However, NMR has a limited sensitivity that can detect only concentrations of 10 µM, or a few nmol, at high fields using cryoprobes (Pan and Raftery 2007). Furthermore, the time to record simple spectra can be 4–5 min, whereas PTR-MS has the capability to measure in a matter of seconds, and while in this study measurements were performed over a few minutes, with the implementation of auto-sampling methodology samples could be measured much faster.

PTR-MS has shown itself capable of revealing considerable shifts in the expression of several urine volatiles. These shifts could be analyzed very rapidly. Fast analyses could be coupled to dedicated multivariate statistical methods, which have already proved their merits in metabolomics, and do so again in this study. Thereby, this technology could be employed in large-scale public events. Specifically those events which focus on diet and physical exercise and in which a large number of individuals can be monitored non-invasively within a time-frame that would allow variations in their physiological states to be observed.

## Concluding remarks

Multivariate analysis allowed for discrimination of before and after exercise for all three groups and on three out of 4 days. The advantage of using multivariate analysis in highlighting 12 ions from 33 is shown clearly by this study. Acetic acid in urinary headspace is identified as a significant marker for exercise effects induced by walking. An increase in acetic acid is observed after exercise for all days, and all groups. The potential to use acetic acid in urine to monitor exercise effects is exhibited and may have particular application in conjunction with PTR-MS for monitoring the effect on participants in a mass participation exercise event. Analysis of acetone concentration with univariate tools revealed different information when compared to breath as a function of exercise; making known an interesting effect of time over the 4 days. Breath samples provide insight into metabolic changes at the moment the sample is taken, whereas metabolites in urine samples result from a longer term diffusion process. This difference in timing may allow complementary information to be obtained.

## Electronic supplementary material

Below is the link to the electronic supplementary material.

Supplementary material 1 (DOCX 224 kb)
