# Supplementary material for: Changes in urine headspace composition as an effect of strenuous walking
Source: Metabolomics. 2015 May 31;11(6):1656–66. doi: 10.1007/s11306-015-0813-8 (PMC4605988; doi:10.1007/s11306-015-0813-8)
Supplement: Supplementary file 1 — Supplementary material 1 (DOCX 224 kb) [file 11306_2015_813_MOESM1_ESM.docx]

Figure 1S: The repeatability of the measurement technique is shown in a) for four cuvettes measured sequentially and containing 10 mL of urine from the same original sample, contrasted with b) showing 3 cuvettes, each containing 10 mL samples from different individuals. The ions shown are *m/z* 43 (acetic acid (fragment)), *m/z* 59 (acetone), *m/z* 61 (acetic acid), *m/z* 73 (succinic acid (fragment)/ hexanoic acid (fragment)).
